# Health outcomes, education, healthcare delivery and quality – 3055. Relation between gastroesophageal reflux disease and asthma

**DOI:** 10.1186/1939-4551-6-S1-P223

**Published:** 2013-04-23

**Authors:** Daymee Novoa Selles

**Affiliations:** 1 Alergy, Policlinico Lidia y Clodomira. Minsap, Cuba

## Background

The Gastroesophageal Reflux Disease (GERD) is not associated with the increase the acid secretion, but by failure of the antireflux barrier with one basal hipotone of the inferior sphincter of the esophagus that can be because of the medicines like Xanthine, Non-Steroidal Anti-Inflammatory Drugs (NSAIDs), alcohol or anticolinergics ^(1)^. The Asthma and GERD are common medical affections and recent studies show that often they coexist ^(2)^. The GERD paper as leading factor of the asthma is an important subject, In addition, also exists the possibility that the asthma can precipitate the ERGE. This investigation purposed to demonstrate the interrelation between Gastroesophageal Reflux Disease and Bronchial asthma

## Methods

By means of a descriptive and traverse study of the patients with bronchial asthma of the municipality Regla assisted in consultation of allergy of April - June 2011, being applied a survey to all the greater asthmatic patients of 20 years for clinical confirmation of the Gastroesophageal Reflux Disease.

## Results

Demonstrating that 63.8 % of the asthmatic ones present GERD, being more frequents in females (64%) and on the group of 40 to 59 years old, the digestive symptom most significant was the postprandial fullness (94%), and digestive extra the cough (81%). The abundant food is the main factor of risk of the ERGE (69%).

## Conclusions

Most of the asthmatic patients suffer Reflux, being demonstrated the narrow relationship among these pathologies. It exists ignorance that the symptoms can be unchained by the GERD and that this last one is increased by Xanthine, NSAIDs, and abundant foods.

